# Supplementary material for: Realized Heritability, Risk Assessment, and Inheritance Pattern in Earias vittella (Lepidoptera: Noctuidae) Resistant to Dipel (Bacillus thuringiensis Kurstaki)
Source: Toxins (Basel). 2022 Oct 6;14(10):686. doi: 10.3390/toxins14100686 (PMC9610588; doi:10.3390/toxins14100686)
Supplement: Supplementary file 1 [file toxins-14-00686-s001.zip › toxins-1879878-supplementary.pdf]

Table S1. Selection history for DIPEL-SEL *Earias vittella* strain.

Generation	Insecticide	Concentration (μg/mL)	Exposed larvae	Dead larvae	Mortality (%)	Survival (%)
G_1	Dipel	45	875	548	62.63	37.37
G_2	Dipel	80	604	286	47.35	52.65
G_3	Dipel	80	840	358	42.62	57.38
G_4	Dipel	120	640	218	34.06	65.94
G_5	Dipel	200	680	208	30.59	69.41
G_6	Dipel	250	590	139	23.56	76.44
G_7	Dipel	300	320	63	19.69	80.31
G_8	Dipel	300	280	48	17.14	82.86
Average			603	233	34.71	65.29
